# Supplementary material for: Trade-offs between multifunctionality and profit in tropical smallholder landscapes
Source: Nat Commun. 2020 Mar 4;11:1186. doi: 10.1038/s41467-020-15013-5 (PMC7055322; doi:10.1038/s41467-020-15013-5)
Supplement: Supplementary file 1 — Supplementary Information [file 41467_2020_15013_MOESM1_ESM.pdf]

Supplementary Information

Trade-offs between multifunctionality and profit in tropical smallholder landscapes

Grass *et al.*

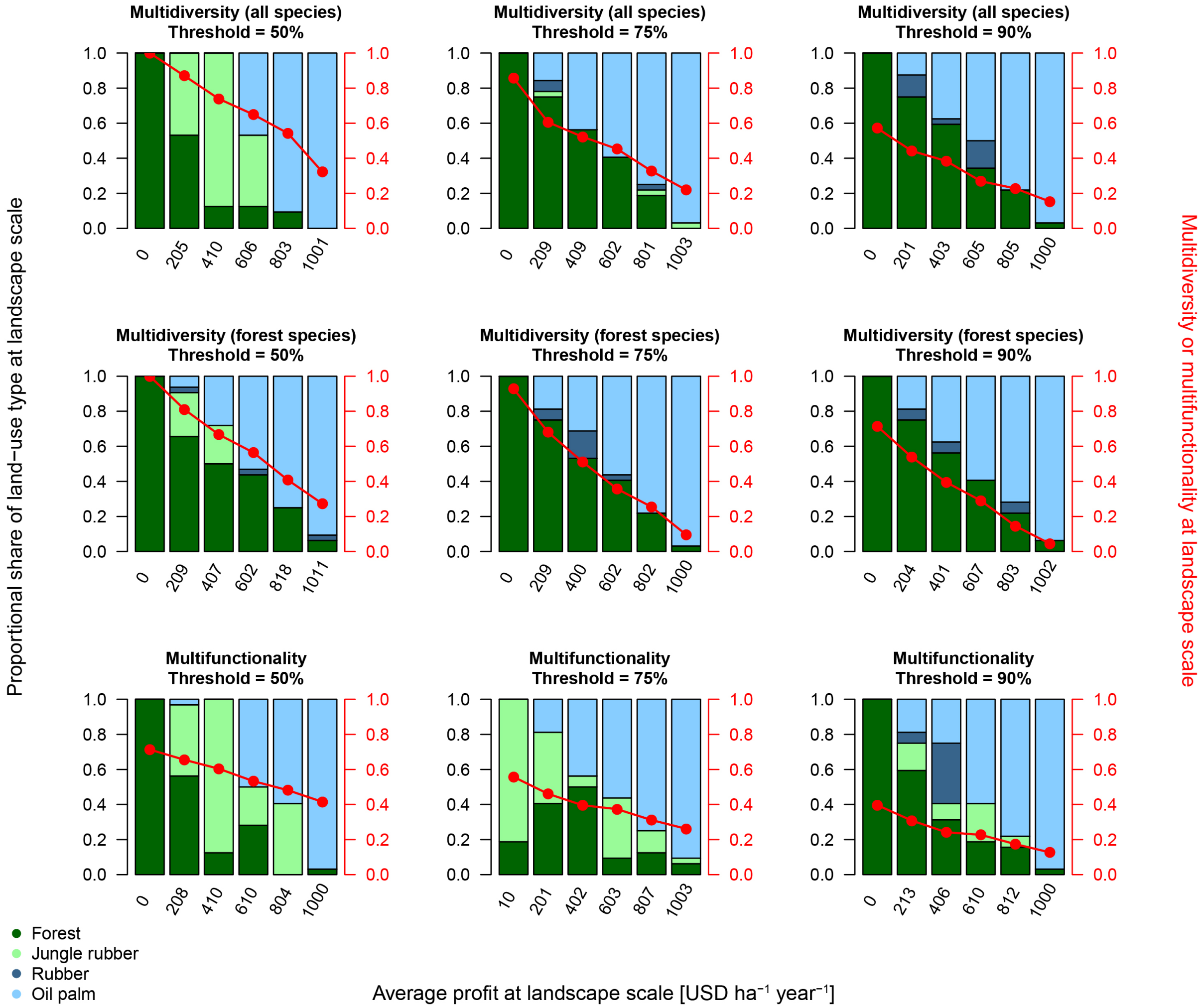

Supplementary Figure 1.
Optimized landscape compositions for multidiversity and multifunctionality with increasing profit expectations and at multiple thresholds used to define multidiversity or multifunctionality. Multidiversity was calculated based on species richness of all species across 14 taxonomic groups and based on species richness of those species that were present in rainforest. Multifunctionality was calculated based on all 10 measured ecosystem functions, with equal weighting of multiple indicators per function. Each bar represents a landscape solution as identified by a genetic algorithm, fed with plot-level information on multidiversity or multifunctionality and profits of smallholder farmers. Colors indicate the composition of landscape solutions, i.e., the proportional share of the four studied land-use systems. Red dots indicate the realized multidiversity or multifunctionality for a given landscape composition, connected by lines to visualize trends with increasing profit expectations. Realized values are scaled between 0 and 1, whereby 1 corresponds to 100% of multidiversity or multifunctionality at the landscape level. *A priori* defined profit expectations: 0; 200; 400; 600; 800; 1000 USD ha^{-1} $year^{-1}$.

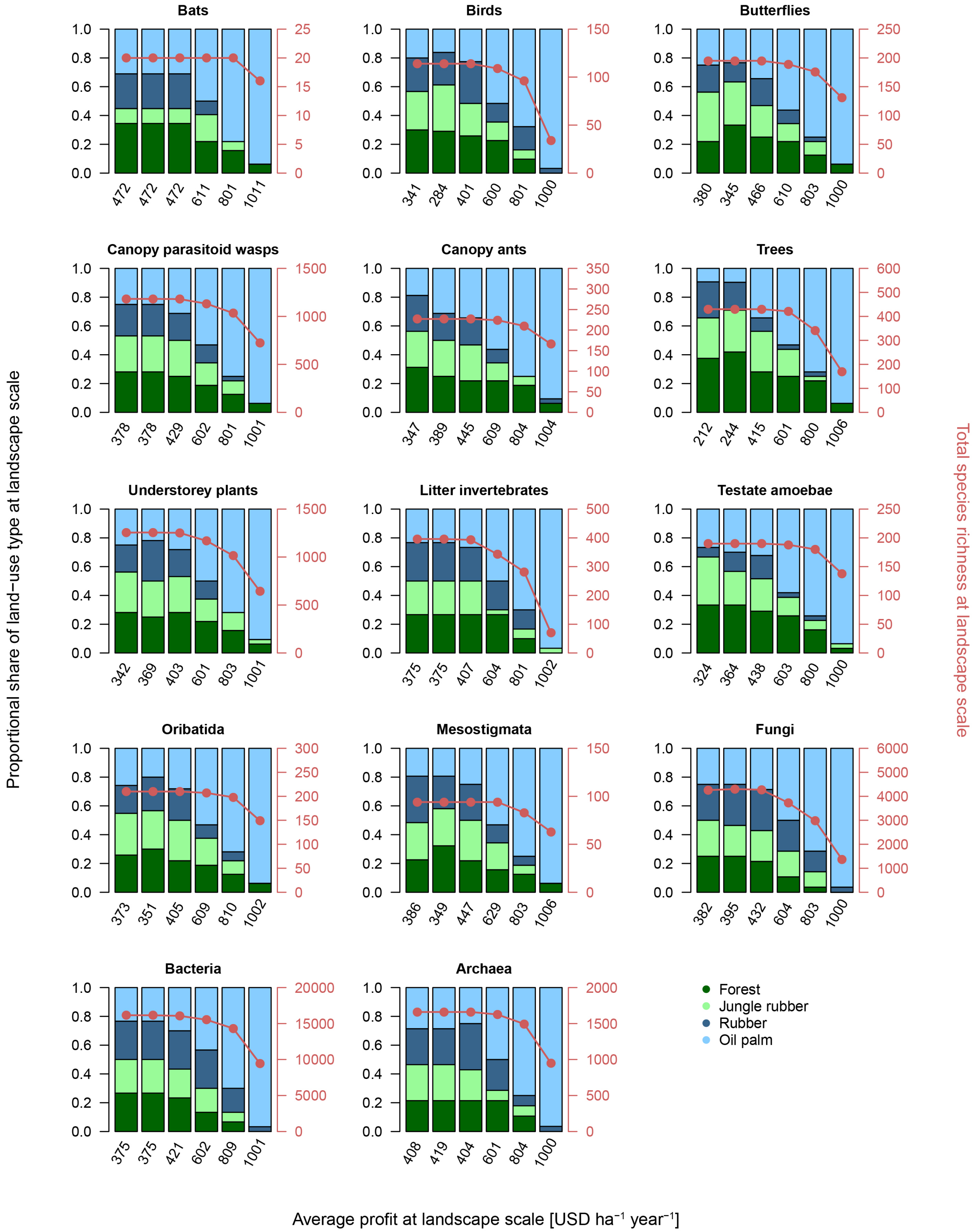

Supplementary Figure 2.

Optimized landscape compositions for highest possible species richness of the 14 studied taxonomic with increasing profit expectations. Each bar represents a landscape solution as

identified by a genetic algorithm, fed with plot-level information on biodiversity and profits of smallholder farmers. Colors indicate the composition of landscape solutions, i.e., the proportional share of the four studied land-use systems. Red dots indicate the realized biodiversity for a given landscape composition, connected by lines to visualize trends with increasing profit expectations. Realized values are scaled between 0 and 1, whereby 1 corresponds to 100% of biodiversity (all sampled species present) at the landscape level. *A priori* defined profit expectations: 0; 200; 400; 600; 800; 1000 USD ha^{-1} $year^{-1}$.

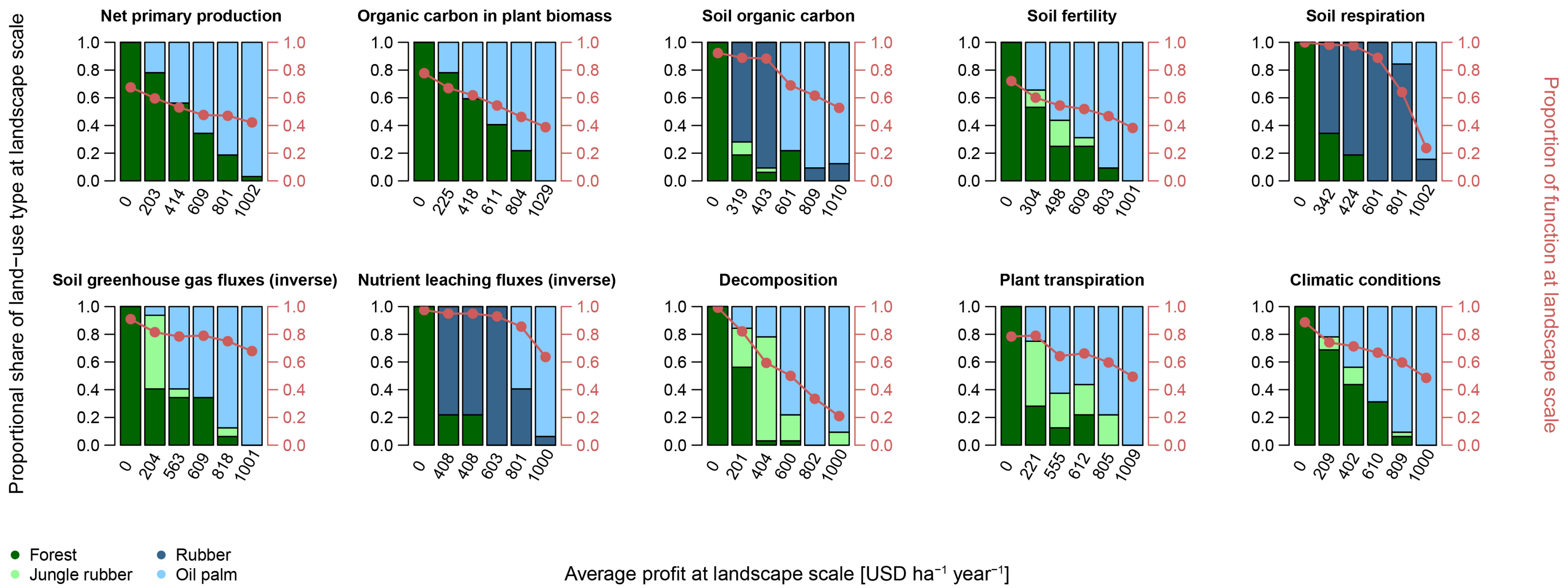

Supplementary Figure 3.

Optimized landscape compositions for highest performance levels of ten ecosystem functions with increasing profit expectations. Each ecosystem function was based on 1–10 equally-weighted indicators, of which the realized performance at landscape scale was simultaneously optimized using a genetic algorithm. Each bar represents a landscape solution as identified by a genetic algorithm, fed with plot-level information on ecosystem functions and profits of smallholder farmers. Colors indicate the composition of landscape solutions, i.e., the proportional share of the four studied land-use systems. Red dots indicate the realized ecosystem function for a given landscape composition, connected by lines to visualize trends with increasing profit expectations. Realized values are scaled between 0 and 1, whereby 1 corresponds to 100% of ecosystem functioning (all function indicators at their maximum) at the landscape level. *A priori* defined profit expectations: 0; 200; 400; 600; 800; 1000 USD ha^{-1} $year^{-1}$.

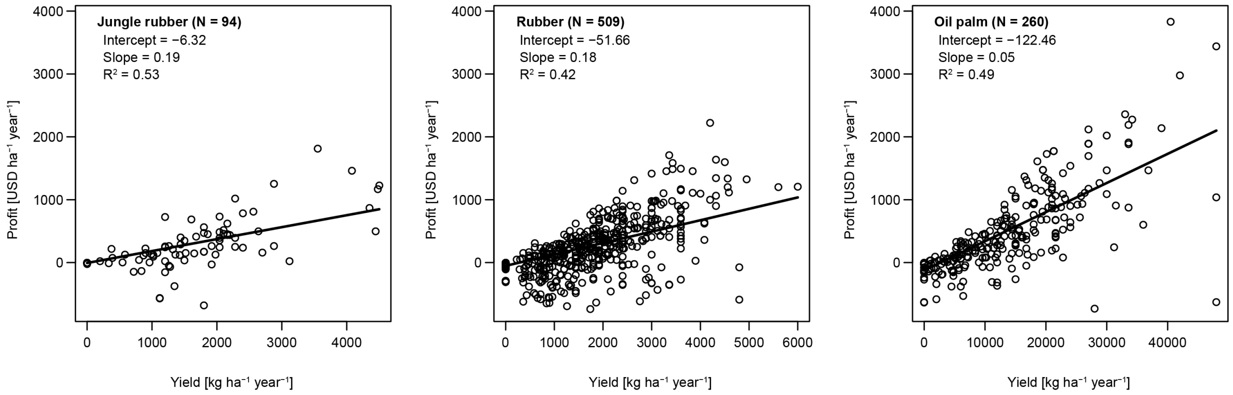

Supplementary Figure 4.

Yield-profit relationships in the studied smallholder land-use systems. Shown are data from household surveys conducted across Jambi province in 2015. At very high yield levels, a few farmers had higher or lower than expected profits (especially for oil palm), which is due to above-average output prices they obtained or above-average input and labor costs.

Supplementary Table 1.

Land-use change (%) in Jambi Province (Sumatra, Indonesia) from 1990 to 2013. Land-use classification inferred from remote sensing.

Land-use	2013						Total 1990	Loss
	Forest	Shrub	Rubber	Oil palm	Agriculture	Other		
**1990**								
Forest	32.50	3.44	5.87	3.53	3.17	0.94	49.46	16.96
Shrub	0.14	5.72	0.07	0.22	0.94	0.15	7.25	1.52
Rubber	1.56	0.55	13.71	1.51	1.66	0.49	19.49	5.78
Oil palm	0.19	0.23	0.15	5.61	0.54	0.16	6.89	1.28
Agriculture	0.05	0.43	0.36	1.15	12.44	0.21	14.65	2.20
Other	0.04	0.00	0.10	0.21	0.06	1.85	2.27	0.42
**Total 2013**	34.49	10.38	20.26	12.24	18.83	3.80	100.00	
**Gain**	1.98	4.66	6.56	6.63	6.38	1.95		

Supplementary Table 2.

Annual profits and agricultural land-use by smallholders in Jambi province, Sumatra, Indonesia based on panel surveys conducted in the years 2012 and 2015. Profit data were derived from plots which were surveyed both in 2012 and 2015 while only including plots with productive trees (435/478 plots). Aggregated land-use was derived from all randomly selected households (658/661 households). Figures in parentheses are standard deviations. The mean values are conditional on cultivation of the crops.

	**Annual profit per ha 2012 (USD)**	**Annual profit per ha 2015 (USD)**
**Oil palm**	968.58 (69.98)	528.90 (52.60)
**Rubber**	958.99 (49.70)	155.82 (43.43)
**Jungle rubber**	663.98 (103.05)	91.65 (182.17)

	**Aggregated land use 2012 (ha)**		**Aggregated land use 2015 (ha)**	
	**Autochthonous villages**	**Transmigrant villages**	**Autochthonous villages**	**Transmigrant villages**
**Oil palm**	371.1	309.12	451.56	344.57
**Rubber**	1,056.6	355.925	1,064.71	366.53
**Jungle rubber**	389.66	77.25	378.11	80.00
**Other perennial crops**	19.21	5.89	21.40	5.78
**Annual crops**	28.08	4.20	28.37	2.58

Supplementary Table 3.
Example calculation of multifunctionality based on the threshold method [1] for three ecosystem functions (F1, F2, F3) measured across 20 study plots. In this example, ecosystem functions contribute to multifunctionality if they locally (at plot level) exceed a 50% threshold, based on the five highest values per function recorded across all study plots. Multifunctionality is then defined as the proportion of functions exceeding this threshold.

Plot	F1	F1 > 50% threshold	F2	F2 > 50% threshold	F3	F3 > 50% threshold	Multifunctionality
1	20	TRUE	0.6	TRUE	202	TRUE	1.00
2	7	FALSE	0.8	FALSE	105	FALSE	0.00
3	13	TRUE	1.2	TRUE	77	FALSE	0.67
4	22	TRUE	0.2	FALSE	188	TRUE	0.67
5	5	FALSE	-0.5	FALSE	140	TRUE	0.33
6	10	TRUE	0.3	FALSE	190	TRUE	0.67
7	6	FALSE	0.1	FALSE	225	TRUE	0.33
8	8	FALSE	0.0	FALSE	123	TRUE	0.33
9	0	FALSE	-0.7	FALSE	55	FALSE	0.00
10	0	FALSE	-0.7	FALSE	206	TRUE	0.33
11	2	FALSE	0.5	FALSE	156	TRUE	0.33
12	4	FALSE	-0.4	FALSE	123	TRUE	0.33
13	7	FALSE	0.2	FALSE	179	TRUE	0.33
14	21	TRUE	0.8	FALSE	147	TRUE	0.67
15	1	FALSE	-1.0	FALSE	170	TRUE	0.33
16	4	FALSE	1.5	TRUE	252	TRUE	0.67
17	6	FALSE	0.5	FALSE	231	TRUE	0.33
18	4	FALSE	0.7	FALSE	201	TRUE	0.33
19	0	FALSE	0.2	FALSE	154	TRUE	0.33
20	6	FALSE	0.2	FALSE	120	TRUE	0.33
**Mean of 5 highest values**	17.2		1.0		223.2		
**50% of this mean**	8.6		0.5		111.6		

Supplementary References

1. Byrnes, J. E. K. *et al.* Investigating the relationship between biodiversity and ecosystem multifunctionality: challenges and solutions. *Methods Ecol. Evol.* **5**, 111–124 (2014).
