# Supplementary material for: Trade-offs between multifunctionality and profit in tropical smallholder landscapes
Source: Nat Commun. 2020 Mar 4;11:1186. doi: 10.1038/s41467-020-15013-5 (PMC7055322; doi:10.1038/s41467-020-15013-5)
Supplement: Supplementary file 3 — Reporting Summary [file 41467_2020_15013_MOESM3_ESM.pdf]

nature research

Corresponding author(s): Dr. Ingo Grass

Last updated by author(s): Dec 30, 2019

Reporting Summary

Nature Research wishes to improve the reproducibility of the work that we publish. This form provides structure for consistency and transparency in reporting. For further information on Nature Research policies, see Authors & Referees and the Editorial Policy Checklist .

Statistics

For all statistical analyses, confirm that the following items are present in the figure legend, table legend, main text, or Methods section.

n/a	Confirmed	
☐	☒	The exact sample size (n) for each experimental group/condition, given as a discrete number and unit of measurement
☐	☒	A statement on whether measurements were taken from distinct samples or whether the same sample was measured repeatedly
☐	☒	The statistical test(s) used AND whether they are one- or two-sided<br>*Only common tests should be described solely by name; describe more complex techniques in the Methods section.*
☐	☒	A description of all covariates tested
☐	☒	A description of any assumptions or corrections, such as tests of normality and adjustment for multiple comparisons
☐	☒	A full description of the statistical parameters including central tendency (e.g. means) or other basic estimates (e.g. regression coefficient) AND variation (e.g. standard deviation) or associated estimates of uncertainty (e.g. confidence intervals)
☐	☒	For null hypothesis testing, the test statistic (e.g. F, t, r) with confidence intervals, effect sizes, degrees of freedom and P value noted<br>*Give P values as exact values whenever suitable.*
☒	☐	For Bayesian analysis, information on the choice of priors and Markov chain Monte Carlo settings
☒	☐	For hierarchical and complex designs, identification of the appropriate level for tests and full reporting of outcomes
☐	☒	Estimates of effect sizes (e.g. Cohen's d, Pearson's r), indicating how they were calculated

Our web collection on statistics for biologists contains articles on many of the points above.

Software and code

Policy information about availability of computer code

Data collection	Original data were provided by personal emails, stored as .csv-files and imported into R (version 3.5.0) using the R-Studio software
Data analysis	All data analyses were done in R (version 3.5.0) using packages simex (ver. 1.7), genalg (ver. 0.2.0), mgcv (ver. 1.8-24).

For manuscripts utilizing custom algorithms or software that are central to the research but not yet described in published literature, software must be made available to editors/reviewers. We strongly encourage code deposition in a community repository (e.g. GitHub). See the Nature Research guidelines for submitting code & software for further information.

Data

Policy information about availability of data

All manuscripts must include a data availability statement. This statement should provide the following information, where applicable:

- Accession codes, unique identifiers, or web links for publicly available datasets
- A list of figures that have associated raw data
- A description of any restrictions on data availability

All relevant data are published on github: https://github.com/ingograss/sumatra_landuse_tradeoffs.

Field-specific reporting

Please select the one below that is the best fit for your research. If you are not sure, read the appropriate sections before making your selection.

☐ Life sciences ☐ Behavioural & social sciences ☒ Ecological, evolutionary & environmental sciences

For a reference copy of the document with all sections, see nature.com/documents/nr-reporting-summary-flat.pdf

Ecological, evolutionary & environmental sciences study design

All studies must disclose on these points even when the disclosure is negative.

Study description	The study focussed on smallholder land-uses on Sumatra, Indonesia. We studied biodiversity, ecosystem functioning and profits across the major four land-uses that characterize the region: forest, jungle rubber, rubber and oil palm. Each land-use was studied in 8 replicate 50 m x 50 m plots. Complemented by panel surveys of 700 smallholder households, we estimated the profits that farmers derived (based on the yields) from their land-uses. These profits were then related to changes in biodiversity or ecosystem functions on the same study plots, with the aim to identify the shape of the economic-ecological relationships and potential trade-offs or synergies of land-use transitions from less profitable to more profitable land-uses.
Research sample	In total, we sampled 14 taxonomic groups and 37 indicators of 10 ecosytem functions on the study plots. For details, please refer to the method descriptions for each group or function.
Sampling strategy	The number of study sites represented a balance between feasability, statistical power and challenging logistics in the study region on central Sumatra. It was not chosen with a single specific variable in mind but based on the large experience of the group consortiuum (from the ecological, social and economic sciences) in interdisciplinary studies in the tropics.
Data collection	Data were collected between 2012 and 2018 by the great majority of co-authors. Details are described in the methods section.
Timing and spatial scale	The majority of data were collected at the plot scale (50 m x 50 m), some data were collected within subplots and afterwards aggregated for analyses (see specific descriptions for taxonomic groups or functions). Depending on the variable, one or multiple measurements per group or function were taken, corresponding to seasonal dynamics and the group or function in questions. Details are described in the methods section.
Data exclusions	No data were excluded from the analyses.
Reproducibility	This large-scale field study can not be reproduced.
Randomization	Full randomization of samples does not apply because of it being a field study. Stratified sampling was chosen for the household survey to provide non-biased estimates of demographic and economic conditions. Field sites were chosen in such a manner to avoid spatial autocorrelation, yet, ensure their association to villages covered by the household survey. The subplots within the study sites were placed randomly as far as local field conditions allowed this.
Blinding	No blinding was conducted, due to the nature of the field study.

Did the study involve field work? ☒ Yes ☐ No

Field work, collection and transport

Field conditions	Field research for this study was carried out in Jambi Province on the island of Sumatra, Indonesia. This tropical lowland region has experienced massive land-use change and transition over the course of the 20th century and is a showcase of smallholder agriculture in Indonesia. The climate in Jambi's lowlands is tropical humid with two peak rainy seasons around March and December, and a dryer period during July–August.
Location	A map of the study villages (socio-economic surveys) and study plots (biodiversity and ecosystem functioning) has been published in Drescher Remboldt et al. 2016, Phil. Trans. Roy. Soc. Biol. Sci. (Ecological and socio-economic functions across tropical land use systems after rainforest conversion)
Access and import/export	Research complied with the national laws of Indonesia and Germany. The socioeconomic surveys were done with consent of the village leaders, respondents and farmers. PT REKI and Bukit Duabelas National Park granted us access to sites and information. The Indonesian Ministry of Research Technology and Higher Eduction, Ristekdikti, granted the research permits.
Disturbance	Permanent study sites were chosen for the the field studies, and disturbance was kept to a minimum as far as possible and restricted to them plots themselves as well as light disturbance on paths (e.g. through the rainforest) providing access to the sites.

Reporting for specific materials, systems and methods

We require information from authors about some types of materials, experimental systems and methods used in many studies. Here, indicate whether each material, system or method listed is relevant to your study. If you are not sure if a list item applies to your research, read the appropriate section before selecting a response.

Materials & experimental systems

n/a	Involved in the study
☒	☐ Antibodies
☒	☐ Eukaryotic cell lines
☒	☐ Palaeontology
☐	☒ Animals and other organisms
☐	☒ Human research participants
☒	☐ Clinical data

Methods

n/a	Involved in the study
☒	☐ ChIP-seq
☒	☐ Flow cytometry
☒	☐ MRI-based neuroimaging

Animals and other organisms

Policy information about studies involving animals; ARRIVE guidelines recommended for reporting animal research

Laboratory animals	NA
Wild animals	Mammal species were identified acoustically, visually or using live-traps in the field, and released on site. Arthropods were collected using different sampling devices, and typically had to be killed to be identified in the lab. However, we do not think that this affected their population sizes, as our studies were restricted to the 50 m x 50 m study plots. Fungi, Bacteria and Archaea were extracted from soil samples. For details please see the methods section.
Field-collected samples	Samples were collected, processed and stored depending as described in the methods section.
Ethics oversight	The studies required no ethics oversight. All work was done in accordance with the laws of Indonesia and Germany.

Note that full information on the approval of the study protocol must also be provided in the manuscript.

Human research participants

Policy information about studies involving human research participants

Population characteristics	The main crop grown by the majority of farmers in the study area is rubber. Oil palm was cultivated by 36% of the households in 2015. Around 60% of the oil palm cultivators also produce rubber whereas sample farmers rarely cultivated food crops. Farm size is about 4 ha. Farm size in this context refers to all land that farmers reported as owning, including land under formal and informal tenure. In 2015, household heads were on average 47 years old, stayed 7 years in school while 45% had a migration background. A considerable share of these migrants were related to the Indonesian transmigrasi program.<br>Oil palm requires less labor than rubber, oil palm farmers can save family labor. These labor savings allow oil palm cultivators to increase their farm size (if additional land can be acquired) and/or to spend more time in non-farm economic activities. The income generated from these alternative uses of the saved time can be seen as secondary effects of oil palm cultivation. There is no difference in the availability of family labor (number of adults in the household) between oil palm and rubber farmers. Human capital endowments, which we capture through age and education of the household head, are also similar between the two groups.
Recruitment	All five regencies in Jambi Province located in the tropical lowland areas - Sarolangun, Batanghari, Muaro Jambi, Tebo, and Bungo - were selected purposively. From these regencies, 40 villages in 20 districts were randomly selected. Five additional villageswere selected purposively to allow for interdisciplinary overlaps with other subprojects. After village selection, a complete list of all farm households was compiled in each village together with the village leaders. From these lists, 6 to 24 farm households were randomly selected. The sample size was adjusted to village population size to mitigate possible sampling bias. All households that owned any agricultural land in the last 5 years were included. In total 701 farm households were interviewed. Data were collected in 2012 and 2015 for the same farm households. Between these two rounds, the attrition rate was at 6%. In total 41 households from 2012 could not be re-interviewed in 2015. Outmigration was the major reason for attrition (56% of attrition). Other reasons included refusal to be interviewed again (24%), death or old age (10%). The sample is representative for farming households and was tested for biases due to attrition.
Ethics oversight	The studies required no ethics oversight. All work was done in accordance with the laws of Indonesia and Germany.

Note that full information on the approval of the study protocol must also be provided in the manuscript.
